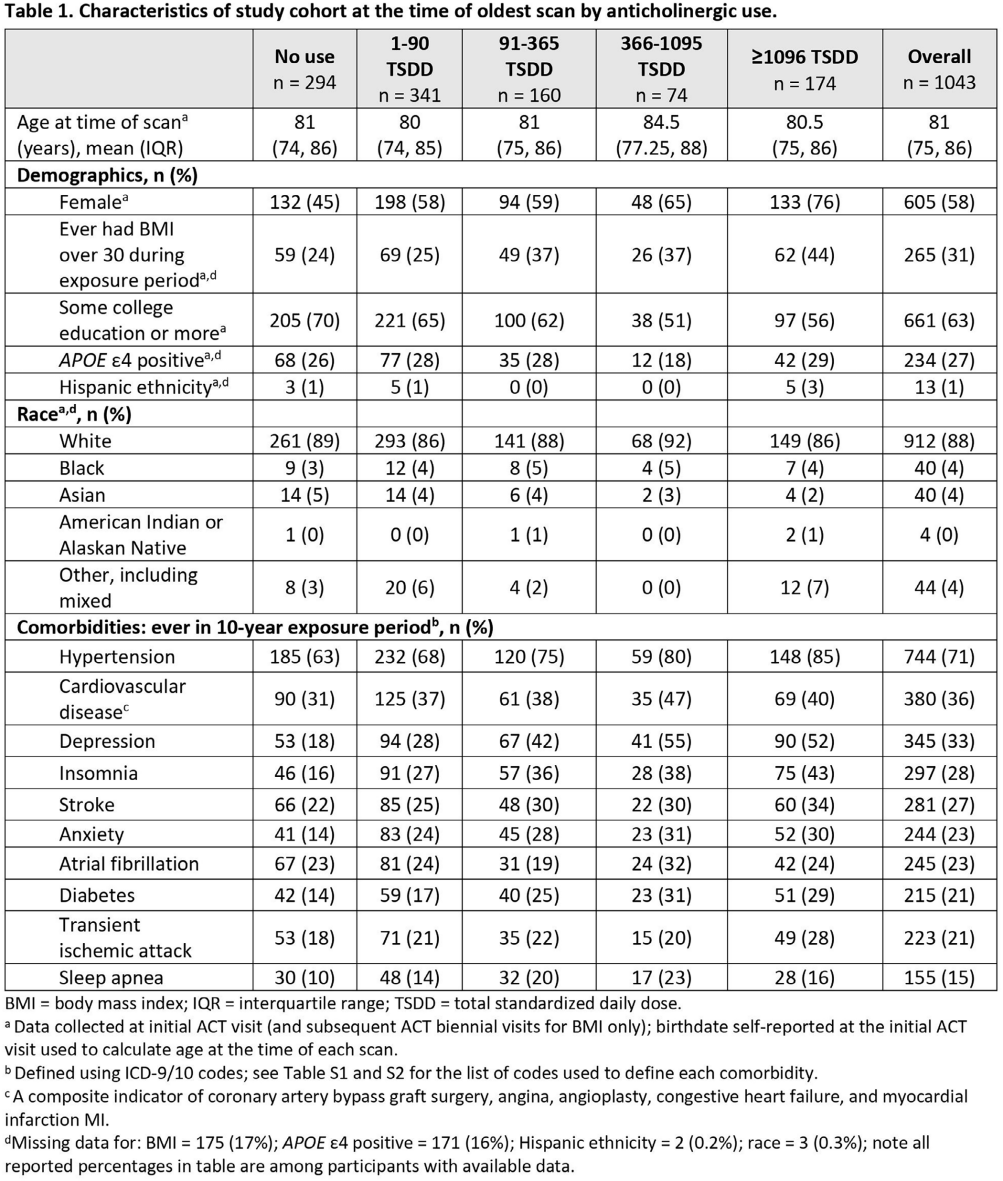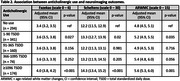# Association Between Cumulative Anticholinergic Use and White Matter Hyperintensity Burden

**DOI:** 10.1002/alz.084407

**Published:** 2025-01-09

**Authors:** Kevin H Li, Chloe Krakauer, Jennifer C Nelson, Paul K. Crane, Jalal B Andre, Patti Curl, Esther C. Yuh, James Ralston, Shelly L Gray, Christine MacDonald

**Affiliations:** ^1^ University of Washington School of Pharmacy, Seattle, WA USA; ^2^ Kaiser Permanente Washington Research Institute, Seattle, WA USA; ^3^ Department of Medicine, University of Washington, Seattle, WA USA; ^4^ University of Washington Department of Radiology, Seattle, WA USA; ^5^ University of California, San Francisco, San Francisco, CA USA; ^6^ University of Washington Department of Neurological Surgery, Seattle, WA USA

## Abstract

**Background:**

Anticholinergic medication use has been found to be associated with higher dementia risk and cognitive decline in older adults. The presence of a biologic pathway through changes to white matter hyperintensities (WMH) remains unclear.

**Method:**

We used the first clinically indicated magnetic resonance imaging (MRI) scan from each participant in the Adult Changes in Thought (ACT) Study—a prospective cohort study within Kaiser Permanente Washington (KPWA)—collected between January 2003 and March 2020 from participants ≥65 years old and with ≥10 years of continuous KPWA enrollment prior to the scan. Our primary cumulative exposure was total standardized daily dose (TSDD) of anticholinergic medications in the 10 years prior to the scan, grouped as non‐use, 1‐90 TSDD, 91‐365 TSDD, 366‐1095 TSDD, and ≥1096 TSDD. Neuroimaging outcomes were common data elements (CDEs) for WMH on MRI scans (Fazekas, Scheltens, Age‐Related White Matter Changes [ARWMC]). We used separate linear regression models for each outcome to estimate adjusted mean values of CDEs in each exposure group and test for differences in mean values.

**Result:**

Of the 1,043 individuals included in analyses, 28% had no use, 33% had 1‐90 TSDD, 15% had 91‐365 TSDD, 7% had 366‐1095 TSDD, and 17% had ≥1096 TSDD. The mean age at the time of the scan was 81 years among a group that was majority female (58%) and White (88%). While most individuals were exposed to antihistamines (38%) among all anticholinergic classes, 65% of TSDD were accrued from antidepressants. Compared to no use, the ≥1096 TSDD group had a higher (worse) adjusted mean Fazekas (4.0 vs. 3.4; p: <0.001), Scheltens (14.3 vs. 12.2; p: <0.001), and ARWMC (5.6 vs. 4.8; p = 0.001). A dose‐response relationship was not found, but adjusted mean outcomes values were observed to be higher (worse) in the 1‐90 TSDD group relative to the no use group.

**Conclusion:**

The highest anticholinergic burden was associated with greater WMH burden among older individuals who received an MRI due to clinical indications. The lack of a demonstrated dose‐response relationship warrants further research into biological mechanisms underlying the link between anticholinergic use and WMH burden.